# Engineering-Oriented Ultrasonic Decoding: An End-to-End Deep Learning Framework for Metal Grain Size Distribution Characterization

**DOI:** 10.3390/s26030958

**Published:** 2026-02-02

**Authors:** Le Dai, Shiyuan Zhou, Yuhan Cheng, Lin Wang, Yuxuan Zhang, Heng Zhi

**Affiliations:** School of Mechanical Engineering, Beijing Institute of Technology, No. 5 South Zhong Guan Cun Street, Haidian, Beijing 100081, China; 3120230370@bit.edu.cn (L.D.);

**Keywords:** ultrasonic characterization, grain size distribution, deep learning, nickel-based superalloy, transfer learning

## Abstract

**Highlights:**

**What are the main findings?**

- Multimodal ultrasonic features with time–frequency encoding and an encoder–decoder model, aided by elliptic spatial fusion, enable grain size distribution prediction for GH4099.
- The method achieves MAEs of 1.08 μm (mean) and 0.84 μm (standard deviation) with a KL divergence of 0.0031, outperforming attenuation- and velocity-based approaches.

**What are the implications of the main findings?**

- Transfer learning calibration rapidly restores accuracy under new input conditions, improving adaptability for practical ultrasonic inspection.
- The framework provides a scalable, low-cost path for accurate, cross-scenario grain size characterization in non-destructive evaluation.

**Abstract:**

Grain size is critical for metallic material performance, yet conventional ultrasonic methods rely on strong model assumptions and exhibit limited adaptability. We propose a deep learning architecture that uses multimodal ultrasonic features with spatial coding to predict the grain size distribution of GH4099. A-scan signals from C-scan measurements are converted to time–frequency representations and fed to an encoder–decoder model that combines a dual convolutional compression network with a fully connected decoder. A thickness-encoding branch enables feature decoupling under physical constraints, and an elliptic spatial fusion strategy refines predictions. Experiments show mean and standard deviation MAEs of 1.08 and 0.84 μm, respectively, with a KL divergence of 0.0031, outperforming attenuation- and velocity-based methods. Input-specificity experiments further indicate that transfer learning calibration quickly restores performance under new conditions. These results demonstrate a practical path for integrating deep learning with ultrasonic inspection for accurate, adaptable grain-size characterization.

## 1. Introduction

Grain size is a key microstructural parameter governing the properties of metallic materials [1]. It directly influences mechanical behavior (strength, toughness, and ductility) as well as electrical and thermal properties and corrosion resistance [2,3]. Therefore, accurate measurement and control of grain size are crucial for materials science, engineering applications, and industrial production [4].

Traditional techniques such as metallography [5] and electron backscatter diffraction (EBSD) [6] are highly accurate but often time-consuming, inefficient, and destructive [7,8]. Consequently, they do not meet industrial requirements such as rapid grain inspection in metallurgy and precise localization of size defects.

Ultrasonic techniques are widely used for material characterization because ultrasonic waves can penetrate materials and are sensitive to microstructural changes [9]. Their non-destructive nature, high spatial resolution, real-time capability, and operational simplicity address the limitations of conventional methods, making ultrasound an increasingly attractive approach [10]. As ultrasonic waves propagate through a material, microstructural parameters such as grain size, morphology, and orientation affect signal transmission [11,12]. By extracting and analyzing ultrasonic features, grain size can be measured rapidly and non-destructively [13]. Current approaches can be broadly grouped into physics-based models and data-driven machine learning methods.

Physics-based methods include the attenuation method [14], ultrasonic velocity method [15,16], and center-frequency method [17]. These approaches derive grain size from propagation characteristics by analyzing attenuation, phase velocity, and spectral features. Although theoretically accurate, their results depend strongly on model assumptions and material specificity, limiting general applicability across materials and process conditions. Moreover, existing theory cannot fully exploit the rich information in ultrasonic signals, and no general analytical formula is available for grain size distribution characterization [18].

Data-driven approaches learn a mapping between grain size and ultrasonic features to enable prediction [19]. Compared with physics-based methods, such models can transfer across tasks and support multi-task characterization [20]. Liu et al. [21] used multi-level wavelet decomposition and a multi-channel one-dimensional CNN to characterize grain size distributions. Zhang et al. [22] combined laser ultrasonics with random forest regression using longitudinal-wave velocity and multi-frequency attenuation. Yu et al. [23] employed GA-optimized BP networks with multi-frequency attenuation features, and Viana et al. [24] extracted time-series features from backscattered signals to classify grain size in ASTM A36 steel. These studies demonstrate the potential of machine learning for grain size prediction, yet their performance is often specific to material type, specimen geometry, and signal conditions. For instance, specimen thickness strongly affects ultrasonic attenuation and velocity [25]. Physical methods typically treat thickness as prior knowledge, whereas many data-driven studies ignore this factor. In automated inspection, the thickness at the measurement point is generally unknown. Therefore, it should be incorporated into prediction targets and constraints to improve generalization across scenarios. In addition, the impact of varying experimental inputs and corresponding mitigation strategies remains underexplored.

This study proposes a deep learning approach for grain size distribution prediction based on multimodal ultrasonic features with spatial coding. First, signal and physical features relevant to ultrasonic grain measurement are extracted from raw data and represented as time–frequency maps. Next, an encoder–decoder model comprising a dual convolutional compression network and a fully connected network is designed for prediction. An elliptic spatial-fusion expectation strategy is then introduced based on the statistical characteristics of metallographic sections. Finally, the method is validated through comparison with traditional approaches.

To address material dependence, the proposed method introduces thickness prediction within the model to learn the latent relationship between grain size and material properties, thereby removing the need for prior thickness measurements in online applications. To study sensitivity to input conditions, signals were collected using probes at three excitation frequencies. Different dataset combinations were constructed to evaluate input-specific generalization, and a transfer learning strategy was proposed to provide practical calibration for new scenarios.

## 2. Materials and Methods

### 2.1. Experiment

The experimental material was a nickel-based superalloy (GH4099) commonly used in aerospace additive manufacturing. Its chemical composition is listed in Table 1.

To obtain ultrasonic signals and corresponding grain size data for each propagation region, we leveraged the fact that ultrasonic acquisition is non-destructive, simple, and low-cost relative to metallographic measurements [26]. The experiment first collected high-spatial-resolution C-scan signals on the sample surface using a customized water-immersion ultrasonic scanning system. The inspection was conducted in pulse-echo mode using a high-frequency focused transducer. The focal point was set at the sample surface to ensure optimal spatial resolution for grain boundary interaction. Several grid points were then selected as centers of metallographic sections, and the metallographic results were used as grain size labels for the nearby ultrasonic signals. The signal acquisition and metallographic procedures are described below.

#### 2.1.1. Ultrasonic Signal Acquisition

**Figure 1:**
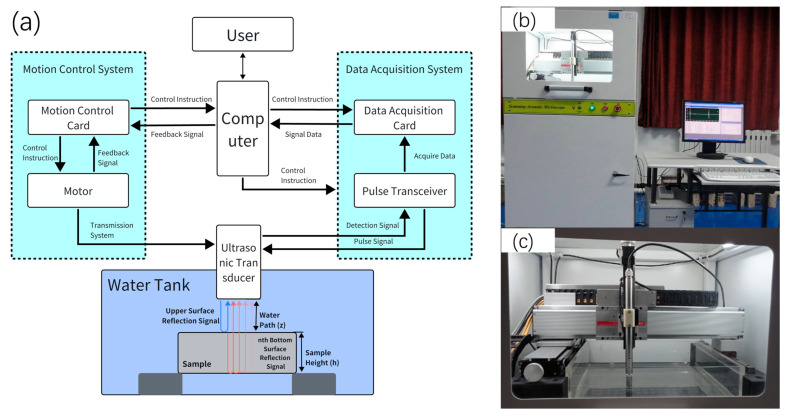
Ultrasonic C-scan detection system. (**a**) Schematic diagram of ultrasonic C-scan signal collection (Transparency represents the process of signal attenuation). (**b**) Equipment control cabinet and computer. (**c**) Three-degree-of-freedom scanning motion system.

The signal acquisition platform was a self-developed ultrasonic C-scan detection system. As shown in Figure 1, it includes a three-degree-of-freedom motion stage, a Panametrics 5900PR pulser/receiver (Olympus Panametrics, Waltham, MA USA), and a Galil DMC-21 × 3 motion controller (Galil Motion Control, Inc., Rocklin, CA, USA). Piezoelectric probes with center frequencies of 5 MHz, 10 MHz, and 20 MHz were used. Notably, while the 20 MHz probe was utilized to enhance sensitivity to smaller grains, the ultrasonic waves in the nickel-based superalloy (GH4099) undergo significant frequency-dependent attenuation and Rayleigh scattering. This physical phenomenon leads to a distinct downward shift in the center frequency for the back-wall echoes, resulting in the effective signal energy being concentrated within a lower frequency band than the nominal probe frequency.

To collect multiple back-wall echoes in a single test and reduce interference from reflections at the tank bottom, a spacer of a parallel material was placed under the specimen so that most of the sample center had a water path above the tank bottom; thus, bottom reflections did not interfere with the effective echoes during a single excitation. The scan step size was 0.5 mm, the acquisition window per pulse was 25 μs, and the sampling rate was 100 MHz.

#### 2.1.2. Material Grain Size Distribution Measurement

**Figure 2:**
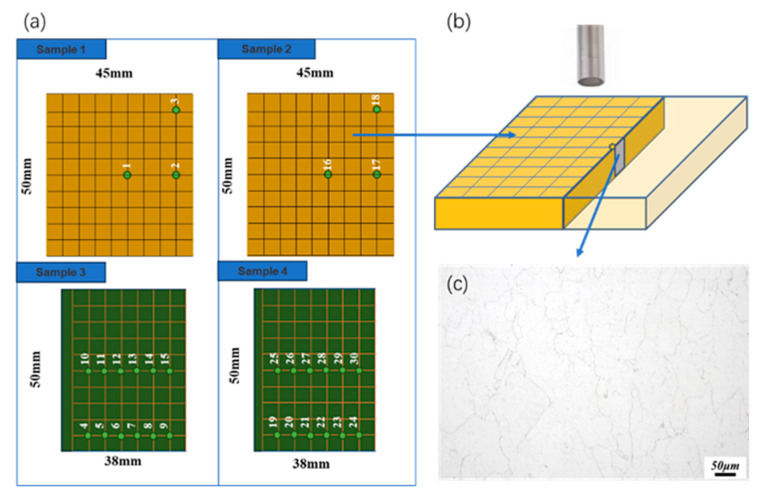
(**a**) Schematic diagram of sample and metallographic test checkpoint. (**b**) Three-dimensional schematic diagram of material metallographic test. (**c**) Checkpoint #1 metallographic diagram.

As shown in Figure 2a, four test blocks from different processing batches were used: two of size 50 mm × 45 mm × 5 mm and two of size 50 mm × 38 mm × 5 mm. A total of 30 checkpoints were selected for metallography. As shown in Figure 2b, the metallographic observation surface was parallel to the ultrasonic incidence direction, and each metallographic block measured 5 mm × 5 mm × 5 mm. Sample preparation included grinding, polishing, and etching. The surface was sequentially polished with 180#, 600#, 1000#, 1500#, 2000#, and 3000# sandpapers, followed by diamond paste to a mirror finish. A mixed solution of 10% HCl and 2% FeCl_2_ was used for etching, and an optical microscope was used for observation. Figure 2c shows the micrograph of checkpoint #1. Grain size from the micrographs was quantified using Image-Pro-Plus for comparison with the prediction mode. Specifically, the software quantifies grain size through a series of steps: initial image enhancement and thresholding to isolate grain boundaries, followed by automated measurements using the intercept method or planimetric method in accordance with ASTM E112 standards [27] to calculate the average grain diameter and area distribution.

#### 2.1.3. Grain Size Distribution Measurement Results

This study comprehensively explored the relationship between the average size and its distribution and ultrasonic signals. Therefore, according to the assumption of lognormal distribution of grains [28], the mean μ, standard deviation $\sigma_{d}$, and mean and standard deviation of the log grain size of each region were calculated. The relationships among them are given as follows:

(1) $$P\left(D\right)=\frac{1}{D\sigma_{d}\sqrt{2\pi }}\exp\left(\frac{{\left(-\ln D-\mu \right)}^{2}}{2\sigma_{d}^{2}}\right)$$

(2) $$\bar{D}=\exp\left(\mu +\frac{\sigma_{d}^{2}}{2}\right)$$

(3) $$\sigma_{D}^{2}=\left(\exp\left(\sigma_{d}^{2}\right)-1\right)\exp\left(2\mu +\sigma_{d}^{2}\right)$$

where D represents the grain size, μ is the mean in the logarithmic normal distribution, $\sigma_{d}$ is the standard deviation in the logarithmic normal distribution, $\bar{D}$ is the mean of grain size, and $\sigma_{D}$ is the standard deviation of grain size.

Using these formulas, the statistical parameters of grain size in each region were calculated (Table 2).

**Figure 3:**
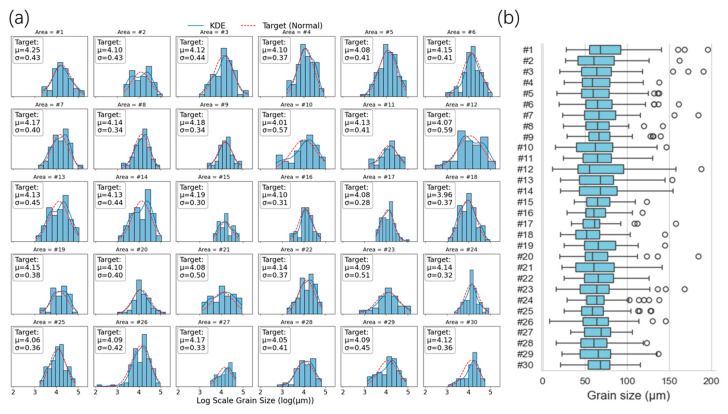
Metallographic measurement results: (**a**) logarithmic grain size distribution histogram; (**b**) grain size distribution box diagram.

The results in Table 2 were visualized to obtain the log grain size distribution histogram (a) and the grain size distribution box plot (b) shown in Figure 3, “cycles” represent possible outliers or deviations. It can be seen from Figure 3a that the grain size of the materials in this study basically satisfies the assumption of log-normal distribution, and the data of different check points have significant distribution differences. The box diagram in Figure 3b further shows the data distribution. It can be seen that the average grain size $\bar{D}$ is distributed in the range of 56–79 μm, mainly concentrated in the vicinity of 65 μm. The larger grain measurement blocks in the same region are marked as outliers (represented by bubbles in the figure), and the standard deviation of grain distribution in different regions is larger. This indicates that the research dataset has a certain degree of generalization diversity and can be used for grain size numerical prediction research.

### 2.2. Model and Method

#### 2.2.1. Data Preprocessing

We employ a deep learning approach for grain size prediction. Unlike traditional machine learning pipelines that emphasize handcrafted feature extraction, deep learning prioritizes information preservation and flexible feature representation (e.g., images or sequences). Theoretically, deep neural networks can approximate arbitrary nonlinear functions [29,30], enabling feature learning directly from raw time-domain signals [31].

However, this conclusion assumes sufficiently large datasets and long training times. In small-sample industrial settings, incorporating prior knowledge through signal preprocessing and model design can compensate for limited data, accelerate convergence, and improve prediction stability [32].

Accordingly, we preprocessed the input signals following three principles: information completeness, noise suppression, and learning adaptability. Effective signal content was preserved, noise was minimized, and features were transformed into representations suitable for deep learning to facilitate efficient feature extraction and pattern recognition.

**Figure 4:**
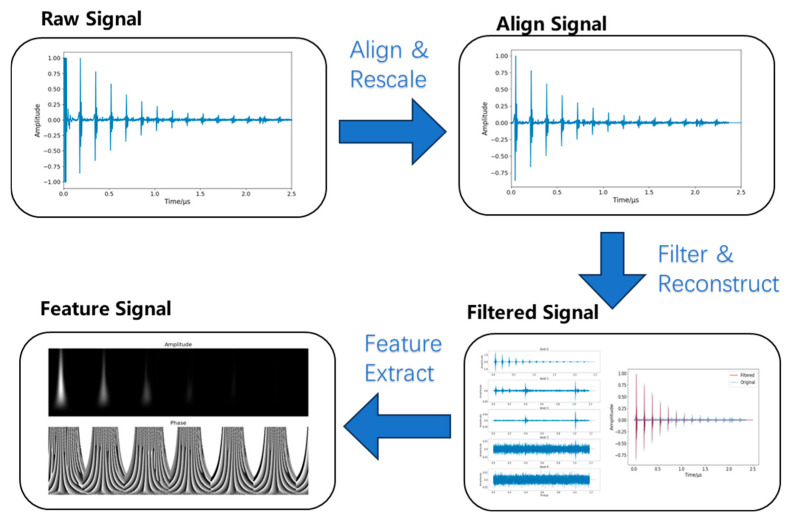
Signal preprocessing and feature extraction pipeline.

Figure 4 illustrates the full preprocessing pipeline. The raw signal shows periodic attenuation; the first pulse is the longitudinal wave reflected from the surface, and subsequent pulses are reflections from the back wall after propagation through the material. The pipeline aligned echoes, applied band-pass filtering and discrete wavelet denoising, and then used a continuous wavelet transform to extract time–frequency amplitude and phase features. The steps are detailed below.

First, the signal was aligned by shifting the first back-wall echo to the start of the record and normalizing its amplitude to the maximum value, thereby removing variability in input energy.

**Figure 5:**
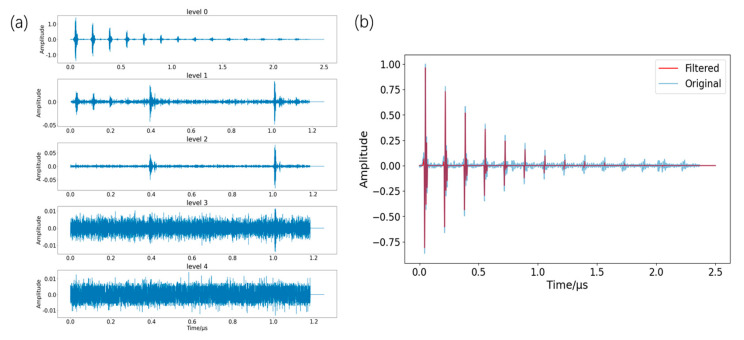
(**a**) db4 discrete wavelet decomposition of each level signal; (**b**) filtering before and after ultrasonic signals.

Next, denoising was performed. A fourth-order Butterworth band-pass filter (1–20 MHz) was employed to eliminate out-of-band noise. Its upper cutoff frequency of 20 MHz was specifically chosen to preserve the full spectrum of the downshifted 20 MHz probe signals while simultaneously suppressing high-frequency electronic noise, which was subsequently followed by soft-thresholding processing with a db4 wavelet at a threshold value of 0.15. As shown in Figure 5a, components above level 0 were treated as noise: levels 1–2 corresponded to shock/echo noise, while levels 3–4 represented random noise. The reconstructed signal in Figure 5b shows a markedly improved signal-to-noise ratio, indicating effective separation of signal and noise.

Finally, because grain size characterization depends on time–frequency features such as attenuation, center frequency, and ultrasonic velocity, we used a time–frequency map to integrate these features. The short-time Fourier transform (STFT) is common but requires window selection, adapts poorly to varying signals, and provides low resolution for high-frequency components [33].

Wavelet transform provided multi-resolution time–frequency analysis with adaptive resolution, offering better high-frequency resolution. In this study, the complex Gaussian wavelet (cgau8) was selected because its complex-valued nature allows for the simultaneous extraction of amplitude and phase information. Physically, the amplitude channel directly represented the frequency-dependent attenuation of the ultrasonic energy, while the phase channel provided precise information regarding the propagation time and velocity shift. Unlike real-valued wavelets (e.g., Daubechies or Haar) that collapse these features, the dual-channel encoding of cgau8 enabled the CNN to decouple and learn these two dominant physical metrics independently.

(4) $$W_{\Psi }\left(a,b\right)=\int_{-\infty }^{+\infty }x\left(t\right)\cdot \Psi^{*}\left(\frac{t-b}{a}\right)\frac{dt}{\sqrt{\left|a\right|}}$$

where $x\left(t\right)$ is the original signal, $\Psi^{*}$ represents the complex conjugate of the wavelet function, a is the scale parameter controlling dilation, and b  is the translation parameter controlling position.

**Figure 6:**
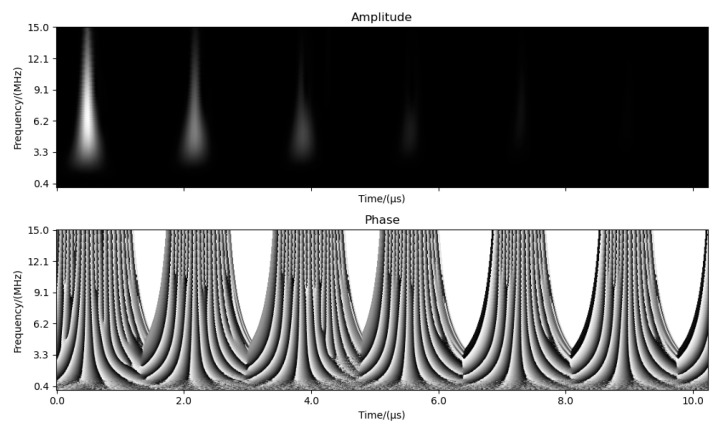
Time–frequency feature map extracted from CGAU8 continuous wavelet transform.

The scale parameter satisfies the following relationship:

(5) $$a\times f=F_{s}\times wcf$$

where $F_{s}\;$ is the sampling frequency and wcf is the wavelet center frequency, determined by the wavelet family and independent of the data itself; 1024 frequency points from 1 to 15 MHz were sampled uniformly. The 1–15 MHz range for continuous wavelet transform (CWT) was chosen because experimental analysis revealed that the signal-to-noise ratio (SNR) for back-wall echoes across all three probes was highest within this interval. Frequencies above 15 MHz for the 20 MHz probe were found to contain predominantly scattering noise and negligible coherent echo energy due to the material’s ‘low-pass’ filtering effect during propagation. The first 1024 time points were then used to form a 2 × 1024 × 1024 wavelet time–frequency diagram with amplitude and phase channels. The resulting map is shown in Figure 6. The amplitude diagram provides good time-domain and frequency-domain resolution and captures the center frequency and attenuation process. This image data can be combined with CNNs for training, and it satisfies the three principles of signal completeness, noise suppression, and learning adaptability.

#### 2.2.2. Dataset Generation

**Figure 7:**
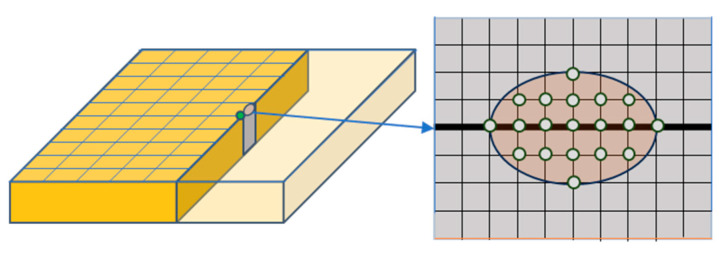
Schematic diagram of ultrasonic signal elliptical space sampling with metallographic section (black line) as the long axis.

Thirty grain-sample points were measured. Because the metallographic area (5 mm × 5 mm) is larger than the ultrasonic scan grid (0.5 mm step), we expanded the dataset by constructing ellipses centered at each metallographic section: 7 and 5 sampling points (corresponding to 3 and 2 mm, respectively) were used as the major-axis lengths (Figure 7). In total, 19 scan points were collected per metallographic position, yielding 570 signals. To integrate the multi-frequency data, signals from the 5 MHz, 10 MHz, and 20 MHz probes were treated as independent observations within a unified dataset. Each A-scan signal, regardless of the source probe, was transformed into an identical 2 ×1024 ×1024 time–frequency feature map, allowing the deep learning model to learn consistent cross-frequency mapping relationships between the ultrasonic backscatter/attenuation patterns and the grain size distribution, rather than relying on frequency-specific features. Combined with data from three probe frequencies, 1710 samples were obtained after time–frequency feature extraction. In this study, we assumed that there was no significant change in the grain distribution near a single sampling point and that the overall grain distribution was relatively uniform.

#### 2.2.3. Deep Learning Model for Grain Size Characterization

The proposed network has two modules: ultrasonic signal encoding and material-property prediction. Accordingly, we design a deep learning architecture consisting of a fully convolutional time–frequency encoder and a fully connected decoder. The network structure is described below.

**Figure 8:**
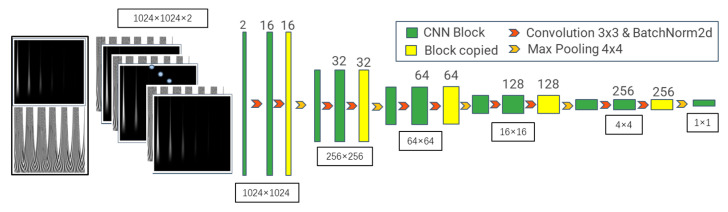
Fully convolutional ultrasonic time–frequency feature encoder network.

Convolutional neural networks (CNNs) are widely used for image representation learning [34]. They capture local image features and, for time–frequency inputs, can extract center-frequency patterns. However, standard convolutions are spatially shift-invariant [35] and may not capture time-domain causality in ultrasonic signals (e.g., attenuation and velocity), which is critical for grain characterization. To address this, we adopt full spatial encoding inspired by U-Net [36], progressively compressing the pixel space, fusing information across scales, and representing signal features through channels. With full compression, the model attains a global receptive field and captures correlations across time–frequency regions. The resulting encoder architecture is shown in Figure 8.

Image feature extraction uses a double-convolution block that preserves spatial resolution while enriching channel-wise representations. The pixel space is then down-sampled using a pooling layer.

The convolution mapping is given by:

(6) $$H_{\mathrm{out}}=\frac{H_{\mathrm{in}}\;+\;2P-K}{S}\;+\;1$$

(7) $$W_{\mathrm{out}}=\frac{W_{\mathrm{in}}+2P-K}{S}+1$$

The pooling mapping is given by:

(8) $$H_{\mathrm{out}}=\frac{H_{\mathrm{in}}-K}{S}\;+\;1$$

(9) $$W_{\mathrm{out}}=\frac{W_{\mathrm{in}}-K}{S}+1$$

where K is the pooling window size, S is the stride, and P is the padding size.

The convolution kernel size was set to (3, 3) with stride (1, 1), and the pooling kernel to (4, 4) with stride (4, 4), reducing spatial resolution to one quarter. A double-convolution block followed by pooling constitutes one compression-sensing block. Stacking these blocks ultimately compresses the spatial map to a single-feature scalar. The compression ratio per block can be adjusted (typically a power of two). Here it is set to 4 based on input size and sample count to avoid excessive training difficulty or insufficient encoding capacity.

The current architecture compresses the 2×1024 ×1024 time–frequency representation into a single feature scalar to ensure a global receptive field, allowing the model to capture the integral attenuation and scattering characteristics across the entire propagation history. This design effectively mitigates overfitting on the current industrial dataset by focusing on the most dominant physical features. For more complex material organizations with higher-order heterogeneity, the encoding capacity could be further enhanced in two ways: (1) Channel Expansion—Transitioning from a single scalar to a high-dimensional latent vector (e.g., 1 ×64 or 1 ×128) to preserve multi-scale structural information; and (2) Variational Constraints—Implementing a Variational Autoencoder (VAE) framework to impose statistical constraints (such as KL-divergence) on the encoding layer. This would ensure that the latent space follows a specific distribution, improving the robustness and physical interpretability of the extracted features under extreme microstructural conditions.

#### 2.2.4. Grain Size Characterization Model

Based on the encoded features, we design a fully connected network to represent material properties. This stage captures the mapping from ultrasonic signals to material features and the correlations among features in the latent space. Incorporating physically meaningful constraints can improve generalization and convergence.

In this study, the direct prediction targets are the mean log grain size and its variance. Physical models indicate that ultrasonic grain characterization depends on material type and local thickness. Representing all material features is complex; therefore, we focus on thickness as a key factor. Because thickness is unknown during automated inspection, it should not be treated as prior knowledge but learned implicitly within the model.

Thickness can be modeled through network design, loss design, or prediction targets. Regardless of approach, thickness implicitly captures signal attenuation and propagation-time dynamics. Here, we adopt a simple strategy: thickness is predicted as an additional output, allowing the network to learn its relationship with grain size in the latent space.

**Figure 9:**
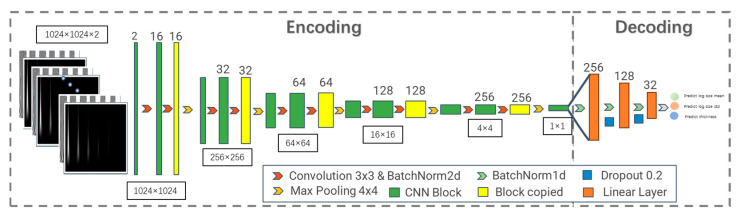
Complete end-to-end encoder–decoder network.

The final network architecture is shown in Figure 9.

When a time–frequency image is input, it is first normalized using two-dimensional batch normalization. The encoder output is flattened and fed into the fully connected network. Batch normalization is applied again to reduce scale differences across channels and accelerate convergence, and a dropout layer is used to improve generalization. The network outputs three values: the mean log grain size μ, the log grain size standard deviation $\sigma_{d}$, and the material thickness h.

#### 2.2.5. Model Training

Grain size distribution prediction is a regression task, so mean squared error is used as the loss. Because the sample distribution is imbalanced, we use a weighted MSE to balance sample contributions. The weighted MSE (WMSE) is defined as follows:

(10) $$\mathrm{WMSE}\;\mathrm{Loss}=\frac{1}{N}\sum_{i=1}^{N}\sum_{j=1}^{M}\left({\left(y_{\mathrm{pred}}^{\left(i,j\right)}-y_{\mathrm{true}}^{\left(i,j\right)}\right)}^{2}\cdot {\mathrm{label}\_\mathrm{weights}}^{\left(i\right)}\right)$$

where N is the Batch Size, M is the number of predicted features; $y_{\mathrm{pred}}^{\left(i,j\right)}$ and $y_{\mathrm{true}}^{\left(i,j\right)}$ represent the predicted value and the true value of the j-th feature of the i-th sample, respectively; and ${\mathrm{label}\_\mathrm{weights}}^{\left(i\right)}$ is the weight of the i-th sample, which will be applied to all features of the sample. In this study task, the weight of the sample with less data was set to 100, and the others were defaulted to 1.

Training used the Adam optimizer with an initial learning rate of 0.001. Cosine Annealing LR was applied with set to 32, the maximum number of epochs was 1000, and early stopping was used. Training stopped if validation loss did not decrease for 200 consecutive epochs, and the model with the minimum validation loss was saved.

## 3. Results

Before training, the dataset was partitioned into training, validation, and test sets strictly at the metallographic region level, rather than the individual signal level. To evaluate the model’s generalization, five specific regions (#5, #9, #17, #25, and #28) were designated as the test set, while the remaining 25 regions formed the training pool. Importantly, probe frequency was not used as a partitioning factor. signals of all excitation frequencies (5, 10, and 20 MHz) originating from a specific region were kept together within the same data split to ensure the model was tested on entirely ‘unseen’ microstructures. A validation set was constructed by randomly sampling 10% from the training pool and 20% from the test set solely for monitoring training performance. While these samples were visible during validation, they did not participate in weight updates (backpropagation), and no significant hyperparameter tuning was performed based on this set, thereby maintaining the validity of the reported generalization performance.

**Figure 10:**
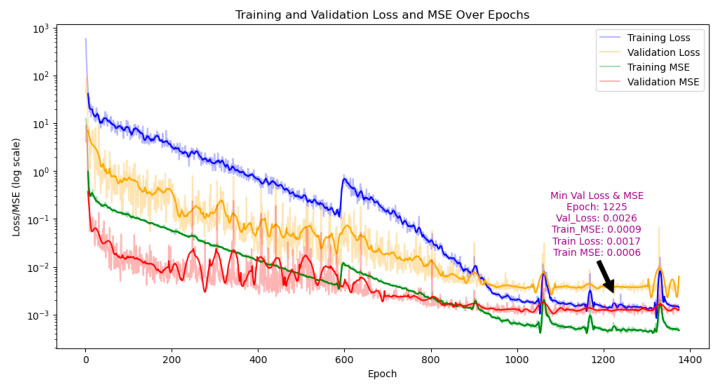
Loss curve during model training.

As shown in Figure 10, the initial loss and WMSE were large. After rapid convergence, the validation loss oscillated briefly and then stabilized after about 800 epochs. At epoch 1225, the validation MSE and loss reached minima of 0.0009 and 0.0026, respectively, and training loss and MSE were 0.0017 and 0.0006. With early stopping, training ended at epoch 1375.

Using this optimal model, the test-set MSE was 0.0010, and the loss was 0.0030, indicating good generalization with limited accuracy degradation on the test set.

**Figure 11:**
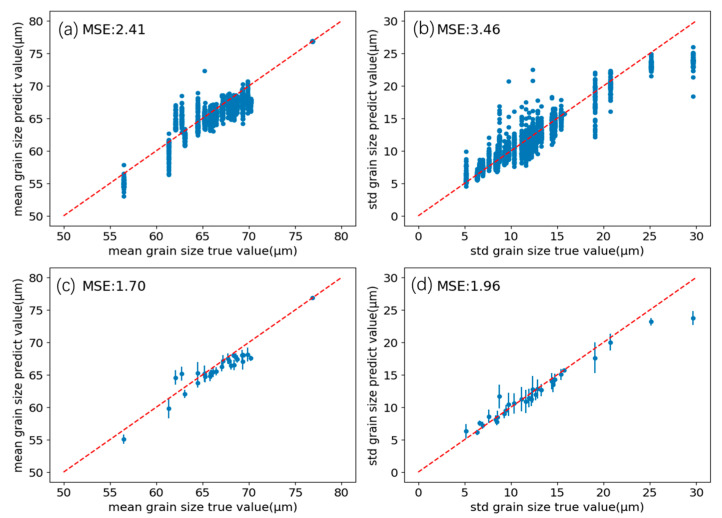
(**a**) Comparison of the predicted value and the true value of the mean grain size before spatial polymerization. (**b**) Comparison of the predicted value and the true value of the standard deviation of grain size before spatial polymerization. (**c**) Comparison of the predicted value and the true value of the mean grain size after spatial polymerization. (**d**) Comparison of the predicted value and the true value of the standard deviation of grain size after spatial polymerization.

To better visualize predicted grain characteristics and thickness effects, the log-normal relationship for log grain size was exponentiated. Mean and variance were computed from Equations (2) and (3), and correlations between predicted and true values are shown in Figure 11a,b. The predicted mean and standard deviation oscillate around y = x (dashed line), with MSEs of 2.41 and 3.46, respectively.

Further, based on the elliptical sampling strategy, assuming that the distance between any point in the elliptical region and the central point is x, there is a spatial distribution function, and the spatial $P\left(x\right)$ fusion expectation expression can be obtained as shown in Equation (11). In this formula, y represents the fusion result, $y_{pred_{i}}$ represents the i-th prediction result of the detection model, and k represents the number of discrete ultrasonic signal detection points in the elliptical region.

(11) $$y=\frac{\sum_{i=1}^{k}y_{pred_{i}}P\left(x_{i}\right)}{\sum_{i=1}^{k}P\left(x_{i}\right)}$$

$P\left(x\right)\;$ can weight the prediction results according to regional correlation. For example, the closer to the central region, the higher the weighting value. In this study, $P\left(x\right)$ was set as a constant term, reducing the expression to a simple average. Calculation showed improved accuracy (as shown in Figure 11c,d): the MSE of the mean prediction becomes 1.70, and the MSE of the standard deviation becomes 1.96, indicating that this spatial fusion strategy helps to improve the prediction accuracy of the model. This result is reasonable because, compared with the spatial resolution of ultrasonic measurement, the grain measurement area is relatively large, and there is not a strict one-to-one correspondence between a single ultrasonic signal and the average grain size. Only by combining ultrasonic multipoint results or refined metallographic measurements can a better numerical relationship between the ultrasonic signal and the material grain be constructed. Based on the assumption that sampling points are uniformly distributed, we adopted a single-point mapping relationship during training and eliminated noise through simple averaging to obtain the final prediction results.

**Figure 12:**
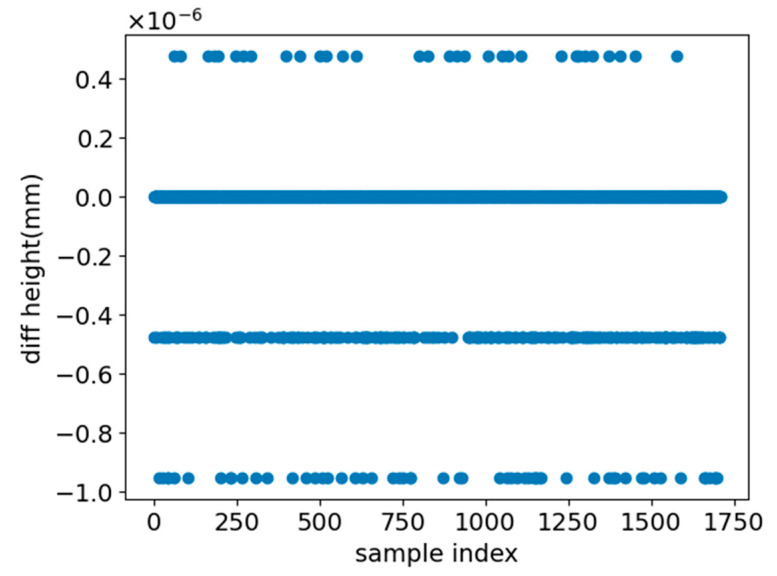
Specimen thickness prediction bias.

Next, the thickness prediction is evaluated via prediction bias. As shown in Figure 12, the predicted thickness closely matches the labels, with errors below 1 × 10^−6^. This suggests the model learns thickness-related constraints that aid characterization.

To quantify differences between predicted and target distributions, we use the Kullback–Leibler (KL) divergence [37]. Equation (12) gives the KL divergence, where values closer to zero indicate greater similarity. Equation (13) provides the KL divergence when the target distribution is normal, expressed in terms of the mean and standard deviation of the target and predicted distributions.

(12) $$D_{KL}\left(p∥q\right)=\int p\left(x\right)\log\frac{p\left(x\right)}{q\left(x\right)}dx$$

(13) $$D_{KL}\left(p∥q\right)=\ln\left(\frac{\sigma_{q}}{\sigma_{p}}\right)+\frac{\sigma_{p}^{2}+{\left(\mu_{p}-\mu_{q}\right)}^{2}}{2\sigma_{q}^{2}}-\frac{1}{2}$$

Due to the asymmetry of the KL divergence, in the equation, we define $p\left(x\right)$ as the target distribution and $q\left(x\right)$ as the predicted distribution. Therefore, the expectation and standard deviation corresponding to $p\left(x\right)$ are $\mu_{p}$ and $\sigma_{p}$, respectively, and the expectation and standard deviation corresponding to $q\left(x\right)$ are $\mu_{q}$ and $\sigma_{q}$, respectively.

The KL divergence is calculated based on Equation (13), and the results are shown together with the grain size. As shown in Table 3, the following information can be obtained from the table:

1. The maximum KL divergence among the 30 samples is 0.0134. Because values below 0.1 indicate high similarity, the results show that all predicted distributions closely match the targets.
2. The model performs better on the training set than on the test set, which reflects normal transfer error. The overall error is within ±2 μm: the mean MAE is 1.08 μm (MRE 1.63%), and the standard-deviation MAE is 0.84 μm (MRE 6.77%), with an average KL divergence of 0.0031. Samples with larger relative errors in standard deviation tend to exhibit larger KL divergence.
3. When samples #12 and #15 are used as training samples, their KL divergence values are large, likely due to large relative errors in predicted standard deviation. In particular, sample #12 shows a large absolute error in distribution prediction. This may arise from a large standard deviation in grain size, which increases local heterogeneity and makes characterization by a single grain-size type insufficient. To address these limitations, future work could replace the simple spatial averaging strategy (Equation (11)) with an attention-weighted fusion mechanism. By assigning higher weights to ultrasonic signals that exhibit higher local entropy or distinct scattering patterns, the model could better focus on anomalous grain structures. Additionally, adopting a finer-grained scanning grid (e.g., reducing the 0.5 mm step) or using multi-scale convolutional kernels in the encoder could help extract localized microstructural gradients that are currently smoothed out by global compression.

**Figure 13:**
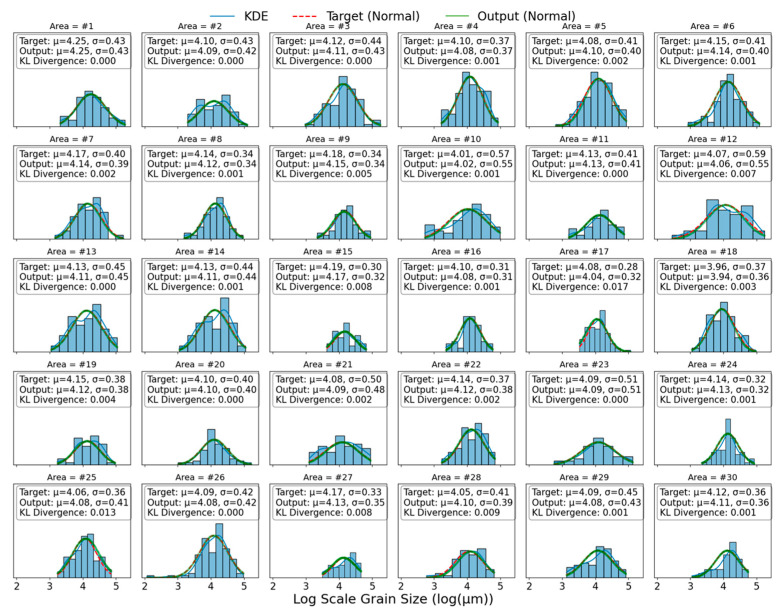
Predicting distribution effect of each regional model based on log-normal distribution.

Further, histograms and kernel density estimation (KDE) curves of the actual distribution of grain size measurements are plotted, and the mean and standard deviation of the true value and predicted value are shown by using the probability density function of the normal distribution. The results are shown in Figure 13. After analysis, it can be seen that for all samples, the overlap effect of the three curves is generally ideal; however, in samples with large KL divergence, some green lines and red lines can be observed to be misaligned. This result strongly demonstrates the effectiveness of KL divergence in characterizing the similarity of distributions.

## 4. Discussion

To further evaluate applicability, we compared the proposed model with conventional methods, examined generalization under input specificity, and discussed transfer learning as a strategy to improve generalization.

### 4.1. Comparison with Other Methods

We compared against the attenuation method and the ultrasonic velocity method. Because these physics-based methods estimate only the mean grain size, we compared mean predictions only.

For both methods, signal preprocessing and denoising were identical to those used for the deep learning approach, after which the data were fitted using the respective physical models.

Firstly, based on the attenuation method, according to the relationship between the wavelength λ and the grain diameter D, there are three common scattering mechanisms [38,39].

(14) $$\alpha \left(\lambda ,D\right)=\left\{\begin{array}{cc} C_{R}D^{3}\lambda^{-4}, & \lambda >>D\left(Rayleigh\right)\\ C_{S}D\lambda^{-2}, & \lambda \approx D\left(Stochastic\right)\\ C_{D}/D, & \lambda <<D\left(Diffusion\right) \end{array}\right.$$

**Figure 14:**
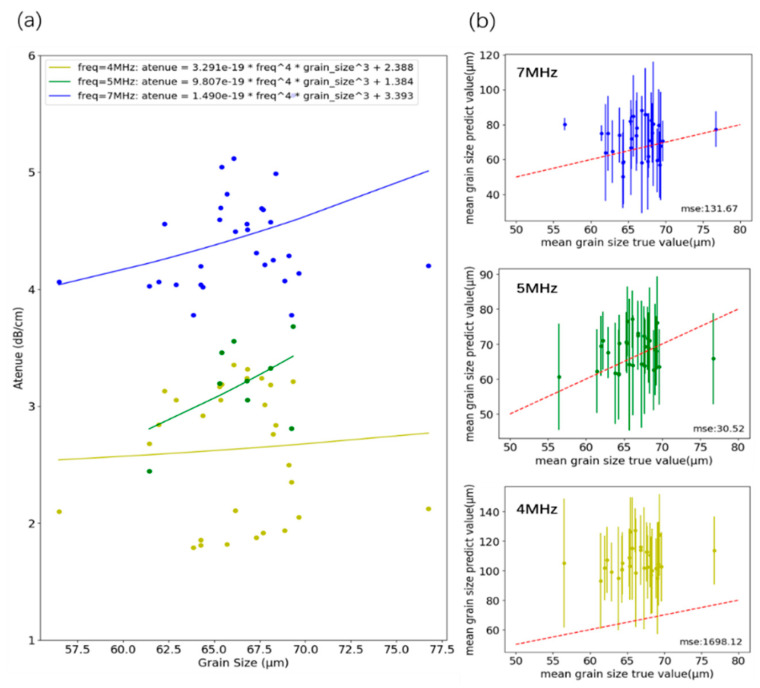
(**a**) Scattering attenuation method fitting; (**b**) average grain size prediction effect of 4 MHz, 5 MHz, and 7 MHz main frequency attenuation model.

In Equation (14), $C_{R}$, $C_{S}$ and $C_{D}$ are material constants. According to the formula and the experimental data of this study, the dominant scattering is Rayleigh scattering. The characteristic frequency band of the signal is identified by FFT and the attenuation of the main frequency is calculated. Then, the fitting grain attenuation formulas of 4 MHz, 5 MHz, and 7 MHz with clear trends are extracted, and the fitting curve and fitting formula are obtained as shown in Figure 14a. Using the fitted formula to calculate the average grain size of all sample signals, the effect is shown in Figure 14b, and the prediction effect of 5 MHz is the best, with an MSE of 30.52.

**Figure 15:**
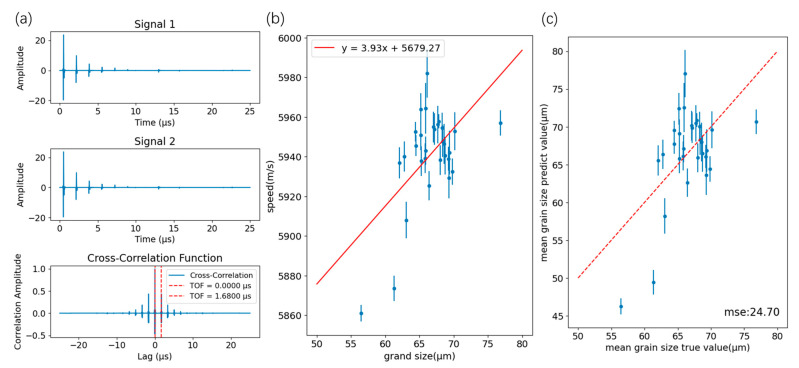
Ultrasonic velocity method. (**a**) Acoustic time calculation based on autocorrelation. (**b**) Linear relationship between ultrasonic velocity and grain size fitting. (**c**) Comparison of the true value of the ultrasonic velocity method with the predicted value.

We then applied the ultrasonic velocity method. As shown in Figure 15a, the arrival time of each pulse was identified using autoregressive analysis; the propagation time was averaged and thickness was computed. Most studies assume a linear relationship between sound speed and mean grain size [15,16]; we fit the data accordingly to obtain the relationship in Figure 15b. Applying this mapping to all signals yields the correlation in Figure 15c, with an MSE of 24.70, slightly better than the attenuation method.

Model performance was evaluated using MSE; the numerical results are summarized in Table 4.

These results show that the deep learning approach offers clear advantages over physics-based methods in characterization capability, prediction accuracy, and scalability.

### 4.2. Input Specificity Influence and Transfer Adaptation Method

Signal acquisition for the same physical process can vary under different experimental conditions. Deep learning models are sensitive to such input specificity. With sufficient computation and model capacity, a model may fit data from a fixed condition, but in practice input conditions are difficult to replicate. Therefore, exploring generalization under varying inputs is both academically and economically valuable.

To investigate this issue, we designed a minimal ablation experiment. The model was trained with 20 MHz and 5 MHz probe signals and tested on 10 MHz signals. Based on the original model, a transfer-training set was constructed by combining 80% of the original test set with 5% of the training set. After about 30 training epochs, the loss converged and training stopped. Table 5 compares performance before and after transfer, showing poor generalization without transfer but rapid convergence and improved accuracy after fine-tuning.

**Figure 16:**
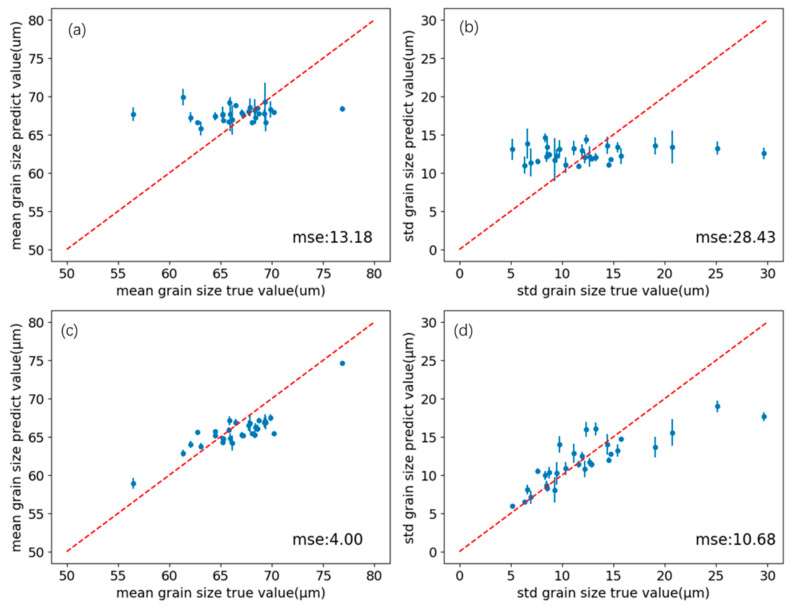
(**a**) Prediction of mean grain size distribution before transfer; (**b**) prediction of standard deviation of grain size distribution before transfer; (**c**) prediction of mean grain size distribution after transfer; (**b**) prediction of standard deviation of grain size distribution after transfer.

For further in-depth analysis, this study visualizes the mean and standard deviation prediction results before and after model transfer, and the results are presented in Figure 16. Before the implementation of transfer learning, the prediction results of the model were highly concentrated around 67 μm, and the numerical maps of the model outputs were very similar for various types of signals. This clearly shows that from the perspective of the initial model, the ultrasonic signals at 10 MHz have high similarity, making it difficult for the model to accurately distinguish subtle differences between different signals, which to some extent reflects that the model has not yet fully grasped the mapping relationship between ultrasonic physical properties and grain size distribution.

However, the model exhibits fast convergence characteristics during the transfer process, and the accuracy is significantly improved after transfer, which can effectively distinguish different signals. This phenomenon confirms that it has effective learning ability for the mapping relationship between the two from the opposite direction. Considering these two seemingly contradictory phenomena, we preliminarily infer that the model has actually learned the mapping relationship effectively. However, due to the lack of data and the unique characteristics of the new signal, the generalization ability of the model in the non-transfer state is limited. The transfer learning method of this experiment can effectively improve the application limitations caused by this problem. In real industrial scenarios, data shortage and equipment differences are common and difficult to avoid. The new data calibration method combined with transfer learning is an effective and promising research idea for deep learning models to be applied in industrial applications.

## 5. Conclusions

This study proposes an end-to-end deep learning method for grain size distribution prediction using multimodal ultrasonic features with spatial coding. By integrating physical-model parameters with deep learning, accurate prediction on GH4099 is achieved. The main findings are as follows:

1. High-Precision End-to-End Prediction: The model encodes material and specimen characteristics within the architecture, enabling end-to-end prediction using only ultrasonic signals. Mean grain size and standard deviation are predicted without prior information on material type or thickness. Across test specimens, errors are within ±2 μm; the mean MAE/MRE are 1.08 μm and 1.63%, and the standard-deviation MAE/MRE are 0.84 μm and 6.77%. A KL divergence-based metric assesses distribution prediction; assuming log-normality, the maximum KL divergence is 0.0167 and the average is 0.0031, indicating high fidelity. Compared with physics-based methods, the proposed approach achieves an MSE of 1.695, substantially lower than 30.518 for the best attenuation model and 24.699 for the velocity method.
2. Multimodal Features Fusion and Network Transferability: The method integrates multiple ultrasonic features (attenuation, center frequency, and acoustic velocity) and applies a spatial fusion strategy aligned with the relationship between grain measurement and ultrasonic resolution. This preserves characterization-relevant information. The encoder–decoder architecture decouples feature extraction from task-specific decoding; the encoder learns robust, multi-frequency features, while the decoder can be adapted through network structure, parameters, and training data to different scenarios, enabling efficient cross-domain transfer.
3. Transfer Learning-Based Model Generalization Analysis: Probe-variation experiments show that, without transfer learning, the original model cannot reliably distinguish grain size distributions for different inputs. With brief transfer training, the model rapidly converges and achieves improved prediction, demonstrating practical applicability through transfer learning calibration and adaptability to scenario-specific conditions.

In summary, the proposed end-to-end approach supports online inspection by combining multimodal feature fusion and a flexible network architecture. The transfer learning strategy improves adaptability to diverse scenarios, providing a practical solution for fast, flexible, and low-cost industrial inspection and highlighting the application potential of deep learning in industrial non-destructive evaluation

## Figures and Tables

**Table 1:** GH4099 alloy chemical composition (in wt%).

C	Cr	Mo	Fe	Ti	Si	Co	Al	W	Ni
0.05	18.53	4.22	1.58	1.20	0.50	6.78	2.10	5.89	Bal.

**Table 2:** Metallographic measurement grain size table.

No.	Average Grain Size (μm)				Log-Normal Distribution (log(μm))	
	$D_{min}$	$D_{max}$	$\bar{D}$	$\sigma_{D}$	μ	$\sigma_{d}$
#1	27.610	195.810	76.755	34.672	4.248	0.432
#2	27.070	162.160	65.712	28.183	4.096	0.428
#3	19.700	190.190	67.688	30.650	4.121	0.439
#4	24.950	137.620	64.274	23.245	4.097	0.371
#5	16.500	137.770	64.273	26.029	4.082	0.412
#6	18.770	160.870	68.869	27.166	4.153	0.410
#7	23.620	184.310	69.631	27.998	4.166	0.401
#8	24.280	142.340	66.170	21.596	4.137	0.344
#9	28.330	139.710	69.232	23.996	4.181	0.341
#10	15.220	146.710	63.847	31.579	4.015	0.571
#11	24.380	129.790	67.303	26.176	4.131	0.409
#12	11.610	187.730	69.106	38.426	4.075	0.594
#13	21.060	153.390	68.228	29.441	4.127	0.450
#14	21.000	154.230	68.371	28.714	4.133	0.441
#15	37.550	123.590	69.330	21.444	4.194	0.302
#16	28.670	117.790	62.917	19.081	4.096	0.310
#17	33.890	157.250	61.431	19.586	4.076	0.284
#18	21.180	144.730	56.481	22.120	3.964	0.375
#19	25.310	145.050	68.096	24.567	4.155	0.375
#20	20.250	184.430	65.289	28.732	4.099	0.397
#21	22.190	141.350	66.807	32.530	4.083	0.500
#22	25.190	126.040	66.826	23.523	4.138	0.368
#23	15.820	167.720	67.595	33.555	4.092	0.514
#24	28.320	137.680	66.081	21.572	4.142	0.315
#25	25.370	128.250	61.956	22.591	4.062	0.363
#26	7.920	145.320	64.390	23.666	4.088	0.425
#27	33.000	106.070	67.772	20.493	4.167	0.326
#28	16.040	123.070	62.269	23.791	4.055	0.412
#29	22.880	137.120	65.360	27.520	4.087	0.446
#30	21.220	115.480	65.415	20.729	4.123	0.363

**Table 3:** Prediction result comparison and accuracy index (MAE—Average Absolute Error; MRE—Average Relative Error; t—target; p—predicted).

No.	Average Grain Size (μm)				Log-Normal Distribution(log (μm))				Indicators				
	${\bar{D}}_{t}$	$\sigma_{D_{t}}$	${\bar{D}}_{p}$	$\sigma_{D_{p}}$	$\mu_{t}$	$\sigma_{d_{t}}$	$\mu_{p}$	$\sigma_{d_{p}}$	$D_{KL}$	MAE		MRE	
										$E_{a\bar{D}}$	$E_{a\sigma_{D}}$	$E_{r\bar{D}}$	$E_{r\sigma_{D}}$
**#1**	76.82	15.73	76.83	15.73	4.25	0.43	4.25	0.43	0.0000	0.02	0.01	0.02%	0.05%
**#2**	65.88	13.24	65.22	12.63	4.10	0.43	4.09	0.42	0.0005	0.66	0.60	1.00%	4.56%
**#3**	67.85	14.43	67.14	14.02	4.12	0.44	4.11	0.44	0.0003	0.71	0.41	1.04%	2.84%
**#4**	64.44	9.50	63.72	9.58	4.10	0.37	4.08	0.37	0.0006	0.73	0.08	1.13%	0.85%
**#5**	64.49	11.94	65.28	11.41	4.08	0.41	4.10	0.40	0.0017	0.79	0.53	1.22%	4.42%
**#6**	69.23	12.70	68.04	11.99	4.15	0.41	4.14	0.40	0.0010	1.19	0.71	1.72%	5.59%
**#7**	69.83	12.18	68.12	11.27	4.17	0.40	4.15	0.39	0.0021	1.70	0.91	2.44%	7.46%
**#8**	66.43	8.35	65.53	8.14	4.14	0.34	4.12	0.34	0.0008	0.90	0.22	1.36%	2.60%
**#9**	69.32	8.52	67.08	8.50	4.18	0.34	4.15	0.35	0.0052	2.24	0.02	3.23%	0.23%
**#10**	65.20	25.11	65.08	23.17	4.02	0.57	4.02	0.55	0.0013	0.12	1.94	0.18%	7.71%
**#11**	67.70	12.36	67.50	12.77	4.13	0.41	4.13	0.42	0.0003	0.21	0.41	0.31%	3.34%
**#12**	70.16	29.65	67.55	23.71	4.08	0.59	4.06	0.55	0.0069	2.62	5.94	3.73%	20.03%
**#13**	68.60	15.41	67.68	15.05	4.13	0.45	4.11	0.45	0.0004	0.93	0.36	1.35%	2.32%
**#14**	68.76	14.75	67.41	14.31	4.13	0.44	4.12	0.44	0.0010	1.35	0.44	1.96%	2.96%
**#15**	69.36	6.62	68.00	7.55	4.19	0.30	4.17	0.32	0.0084	1.36	0.93	1.97%	14.12%
**#16**	63.05	6.38	62.05	6.12	4.10	0.31	4.08	0.31	0.0013	1.00	0.26	1.59%	4.05%
**#17**	61.32	5.15	59.79	6.30	4.08	0.28	4.04	0.32	0.0167	1.53	1.15	2.50%	22.34%
**#18**	56.47	8.51	55.08	7.77	3.96	0.38	3.94	0.36	0.0026	1.39	0.74	2.46%	8.67%
**#19**	68.39	10.33	66.53	10.57	4.16	0.38	4.12	0.38	0.0037	1.86	0.24	2.73%	2.34%
**#20**	65.22	11.11	65.18	11.26	4.10	0.40	4.10	0.40	0.0000	0.04	0.15	0.06%	1.30%
**#21**	67.19	19.06	67.16	17.63	4.08	0.50	4.09	0.48	0.0016	0.04	1.43	0.05%	7.50%
**#22**	67.07	9.73	66.23	10.42	4.14	0.37	4.12	0.38	0.0022	0.84	0.69	1.25%	7.06%
**#23**	68.33	20.69	67.99	20.03	4.09	0.51	4.09	0.51	0.0002	0.33	0.66	0.49%	3.19%
**#24**	66.11	6.92	65.44	7.22	4.14	0.32	4.13	0.32	0.0014	0.67	0.31	1.01%	4.42%
**#25**	62.04	8.73	64.56	11.66	4.06	0.36	4.09	0.41	0.0134	2.52	2.93	4.06%	33.59%
**#26**	65.27	12.90	64.69	12.81	4.09	0.43	4.08	0.42	0.0002	0.57	0.09	0.88%	0.69%
**#27**	68.05	7.65	66.39	8.60	4.17	0.33	4.14	0.35	0.0084	1.66	0.95	2.44%	12.45%
**#28**	62.76	11.58	65.19	10.88	4.06	0.41	4.10	0.39	0.0093	2.43	0.70	3.87%	6.04%
**#29**	65.82	14.50	64.77	13.47	4.09	0.45	4.08	0.43	0.0011	1.05	1.03	1.59%	7.13%
**#30**	65.93	9.31	65.08	9.00	4.12	0.36	4.11	0.36	0.0006	0.85	0.31	1.29%	3.36%

**Table 4:** Prediction of average grain size MSE by different methods.

	Deep Learning	Ultrasonic Attenuation			Ultrasonic Velocity
		4 MHz	7 MHz	5 MHz	
**MSE**	1.70	1698.12	131.67	30.52	24.70

**Table 5:** Before and after transfer comparison table.

	$\bar{D}$ MSE	$\sigma_{D}$ MSE	$D_{KL}$
**Before Transfer**	13.18	28.43	0.0396
**After Transfer**	4.004	10.68	0.0103

## Data Availability

The original contributions presented in this study are included in the article. Further inquiries can be directed to the corresponding author.
